# Survival and prognostic factors in conventional G1 chondrosarcoma

**DOI:** 10.1186/s12957-019-1695-4

**Published:** 2019-09-03

**Authors:** Julian Fromm, Alexander Klein, Andrea Baur-Melnyk, Thomas Knösel, Lars Lindner, Christof Birkenmaier, Falk Roeder, Volkmar Jansson, Hans Roland Dürr

**Affiliations:** 1 Department of Orthopaedic Surgery, Musculoskeletal Oncology, Physical Medicine and Rehabilitation, University Hospital, LMU Munich, Marchioninistr. 15, 81377 Munich, Germany; 2 Department of Radiology, University Hospital, LMU Munich, Munich, Germany; 3 Institute of Pathology, University Hospital, LMU Munich, Munich, Germany; 4 Department of Medicine III, University Hospital, LMU Munich, Munich, Germany; 5 Department of Radiotherapy, University Hospital, LMU Munich, Munich, Germany; 6 CCU Radiation Oncology, German Cancer Research Center (DKFZ), Heidelberg, Germany

**Keywords:** Chondrosarcoma, Low-grade, Surgery, Curettage, Margin status, Recurrence, Prognostic factors

## Abstract

**Background:**

Chondrosarcoma is the second most frequent malignant bone tumor. Grade I chondrosarcoma (syn.: atypical cartilaginous tumor) is classified as an intermediately and locally aggressive neoplasm and typically is treated less aggressively (i.e., by intralesional curettage). Does the data regarding local recurrence (LR) and metastatic disease justify this?

**Methods:**

From 1982 to 2014, 37 consecutive patients with G1 chondrosarcoma had been resected or curetted. The margin was defined as R0 (wide resection) or R1 (marginal resection). All patients were followed for evidence of local recurrence or metastatic disease. Overall and recurrence-free survival were calculated, and various potentially prognostic factors were evaluated.

**Results:**

In 23 patients (62%), the tumor was widely (R0) resected, whereas in 14 patients, (38%) the resection was marginal (R1). Overall survival was 97% after 5 years, 92% after 10 years, and 67% after 20 years. Five-year local recurrence-free survival was 96%. Ten-year local recurrence-free survival was 83%. Local recurrence-free survival showed a significant correlation to margin status but no correlation to location or age. None of the patients with local recurrence died during the follow-up. One patient had metastatic disease at initial presentation, and a further five patients developed metastatic disease during follow-up. Metastatic disease proofed to be a highly significant factor for survival but was not correlated to local recurrence.

**Conclusions:**

There was no significant correlation between the outcome and the primary tumor location. Marginal resection was a risk factor for LR, but there was no significant difference in the overall survival in patients with or without LR. Metastatic disease (16%) was more common than expected from the literature and a significant predictor for poor overall survival.

## Background

Representing more than 20% of all malignant tumors of the bone, chondrosarcoma (CS) is the third most common primary malignant bone tumor following osteosarcoma and multiple myeloma [1]. Chondrosarcomas are most often seen in adult age and are a very heterogenous group with a diverse behavior depending on the histological subtype. The most common subtype is conventional CS. Clear cell CS appears to have the best prognosis while dedifferentiated CS has the worst outcome [2]. For conventional CS, tumor grading and anatomic location are the main predictors of outcome [2–4].

Therapy consists mainly of surgical resection. In critical locations, radiotherapy in high dosage (if applicable) is effective as an adjuvant or as the sole therapy [5] while chemotherapy appears to be less effective [6, 7]. The definition of adequate surgical margins varies within the literature [4]. In high-grade chondrosarcomas, a wide resection is the standard to prevent local recurrence. In low-grade chondrosarcoma, intralesional curettage is commonly used although controversial [8–10]. In a large literature review in 2017, Chen et al. found only 1.2% metastatic disease and no difference in local recurrence with respect to surgical margins [11].

The WHO classification of grade I chondrosarcoma (atypical cartilaginous tumor) as an intermediately and locally aggressive neoplasm implies that there is no or only rarely metastatic disease with low-grade chondrosarcoma [12]. This definition comprises the benign clinical behavior of the lesions, but it is known that even grade I CS carries a risk of metastasis in up to 6% of cases [12].

With intralesional curettage becoming more and more common in low-grade chondrosarcoma, it appears important to take a closer look at the influence of less aggressive (intralesional) surgical margins and outcomes in grade I CS. Does a wider margin prevent metastases or local recurrence in low-grade CS? Is the location of the lesion a predictor of outcome in low-grade lesions as it is in high-grade CS [13, 14]?

The main aim of this retrospective study hence was to have a closer look at this very selective and homogenous group of patients with primary low-grade conventional CS treated at a single tumor center to determine prognostic factors for overall and recurrence-free survival. The secondary aim was a comparison of our data to the literature for quality-of-care reasons.

## Materials and methods

### Patients

From 1982 through 2014, 87 consecutive patients with central chondrosarcoma of the extremities, the pelvis, and the trunk wall were treated at our institution. All patients had a diagnosis of chondrosarcoma based on histological, radiological, and clinical features.

Preoperatively, mainly magnetic resonance imaging (MRI) and in some cases computed tomography (CT) were used to define size and location of the tumor. A CT scan of the chest was obtained to rule out or prove metastatic disease.

### Margins and inclusion criteria

All patients underwent surgical resections. The margin was defined as R0 if a rim of healthy tissue around the lesion was present (wide resection) or R1 if the margins were contaminated as in close resections or curettages. From those 87 patients, 37 showed a low-grade (G1) histology and were classified as atypical cartilaginous tumors. Inclusion criteria for this study were therefore a histology-proven G1 grading in the resected specimen with or without proven metastatic disease.

### Statistical analysis

All patients were followed for evidence of local recurrence or distant metastasis in general by MRI scans and chest x-rays. Due to the long investigation period with considerable changes in the ability to detect small metastases, especially in the lungs, and small local recurrences, the number of metastatic cases as also local recurrences might be underestimated. Subsequently, recurrence-free survival would be overestimated. Therefore, in addition, overall survival is calculated from the time of surgery to last follow-up or death in deceased patients. Overall and recurrence-free survival were calculated by the Kaplan-Meier method. Significance analysis was performed using the log-rank test or the chi-square test. The data analysis software used was SPSS 24®.

## Results

The mean age of the 12 female and 25 male patients was 47.1 years (range 17–84 years). The upper extremity was involved in 4 cases (humerus 2, radius 1, hand 1), and the lower extremity in 23 cases (femur 15, tibia 4, fibula 2, feet 2). Five patients had the lesion in the trunk (scapula 4, ribs 1), and five in the pelvis (4 os ilium, 1 os pubis). Sixteen (43%) showed extraosseous tumor growth.

The median duration of symptoms was 19.8 months (range 0–153 months). Nineteen patients (51%) complained of pain, 3 (8%) complained about swelling, and 5 (11%) suffered from a pathological fracture, and in 6 cases (16%), the lesion was found as an incidental finding. Occasionally, there were neurological symptoms or a loss of the range of motion. Thirty-one patients (84%) had a biopsy taken before surgery. In four cases, the biopsies showed benign cartilaginous lesions, later classified as false negatives. Six patients had undergone intralesional surgery elsewhere with either intramedullary nails or endoprostheses prior to presenting to our institution, and the tumor had been overlooked or underestimated in these cases. One patient had metastatic disease at the time of diagnosis.

In 28 patients, the tumor was resected or curetted and the defect, as necessary, was filled with allogenic bone graft. A reconstruction with megaendoprostheses was performed in 8 (22%), and an amputation in 1 case (2%). In 23 patients (64%), the tumor was widely (R0) resected, whereas in 14 patients (38%), the resection was marginal (R1). There were no R2 resections. Histology showed a conventional low-grade chondrosarcoma in all patients. In pelvic lesions, 66% were marginal resections compared to 92% at the lower extremities and 33% at the upper extremities. There were no marginal resections at the trunk.

In five patients (14%), revisions due to complications had to be performed. The complications included loosening of an endoprosthesis in two, neurological impairment because of a malpositioned osteosynthesis screw, additional and more aggressive tumor resection, and deep wound infection in one case each.

**Fig. 1:**
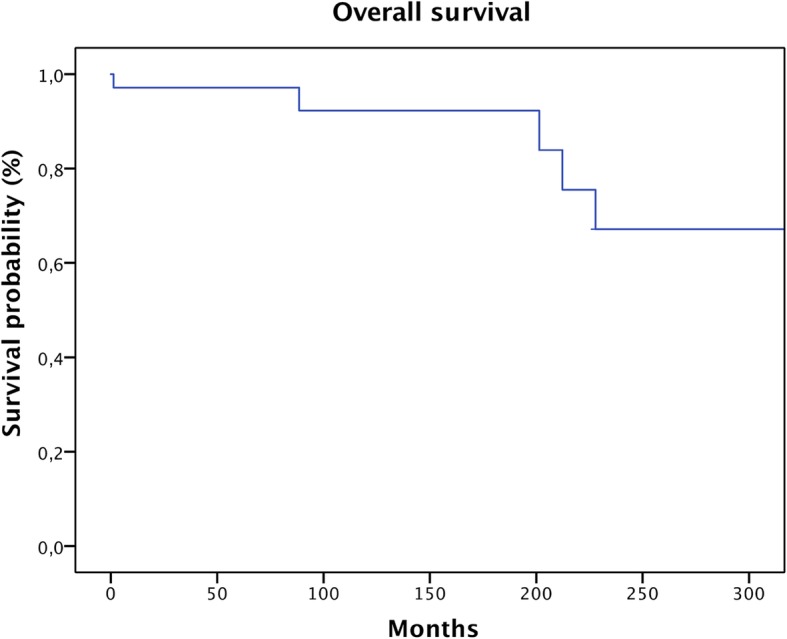
Survival of all patients with conventional G1 chondrosarcoma

The mean follow-up in our series was 127.9 months (range 0–344 months). Only 3 patients had a follow-up time of less than 24 months, and 13 patients (35%) had a follow-up of less than 5 years. Five patients died during the follow-up: one within the first year, one after 7 years, and three after more than 15 years (Fig. 1).

**Fig. 2:**
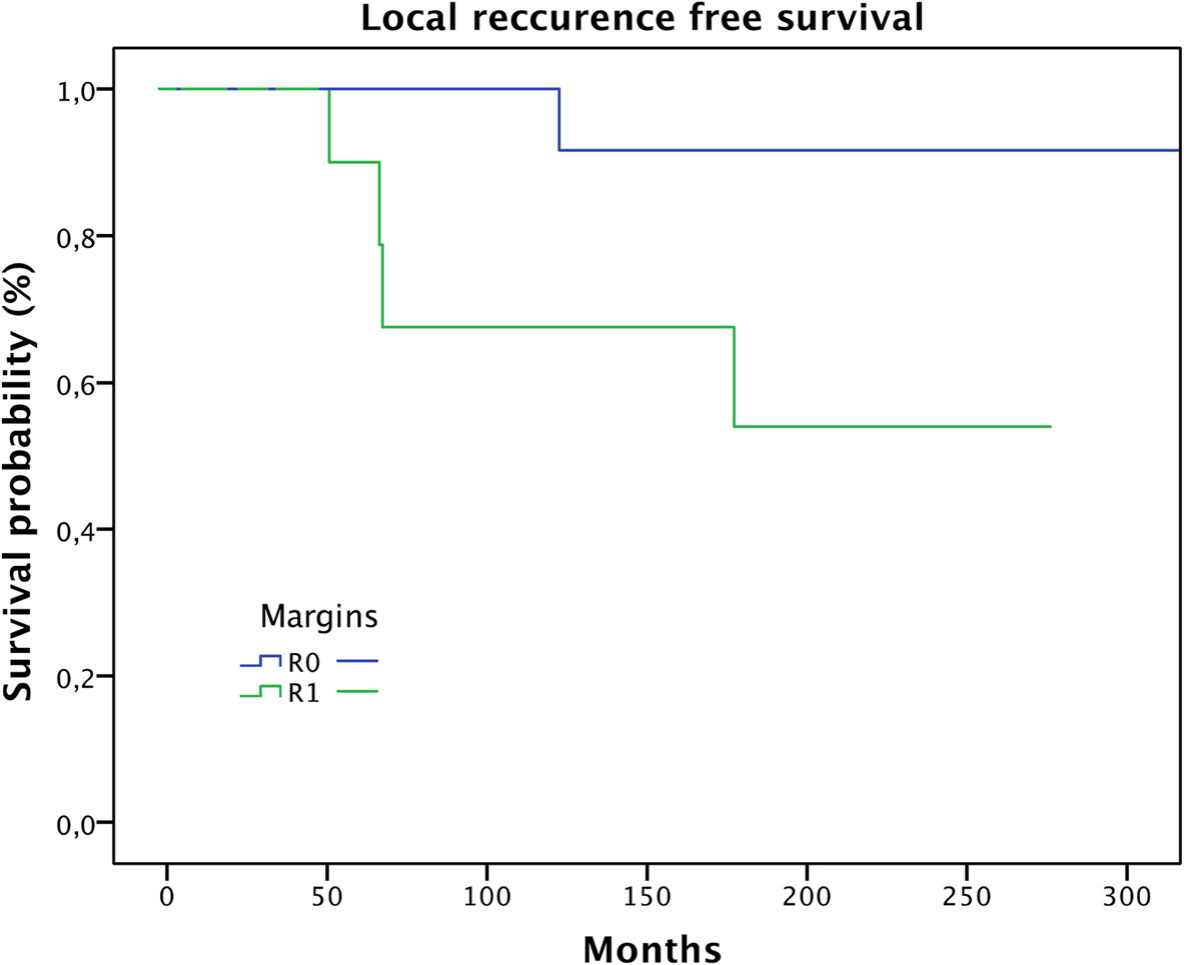
Influence of surgical margins on local recurrence-free survival (*p* = 0.035)

Overall survival was 97% after 5 years, 92% after 10 years, and 67% after 20 years (Fig. 1). Five-year local recurrence-free survival was 96%. Ten-year local recurrence-free survival was 83%. In total, 5 (14%) patients developed local recurrences: only 1 of them during the first 5 years and 4 after 10 years. In our patients, local recurrence-free survival showed a significant correlation with the margin status (Fig. 2; *p* = 0.035) but no correlation with the location or the patients’ age. This was confirmed by means of multivariate analysis (Table 1).

**Table 1:** Local recurrence in relation to surgical margins and tumor location

Local recurrence	No	Yes	*p* value	5-year LRFS (%)	10-year LRFS (%)	15-year LRFS (%)	*p* value
R0	22 (96%)	1 (4%)	0.037	100	92	92	0.035
R1	10 (71%)	4 (29%)		90	79	54	
Upper extremity	4 (100%)	0 (0%)	0.097	100	100	100	0.238
Lower extremity	18 (78%)	5 (22%)		94	71	60	
Pelvis	5 (100%)	0 (0%)		100	100	100	
Trunk	5 (100%)	0 (0%)		100	100	100	

None of the patients with local recurrence (LR) died during the follow-up, and only one showed LR and metastatic disease (MD).

**Fig. 3:**
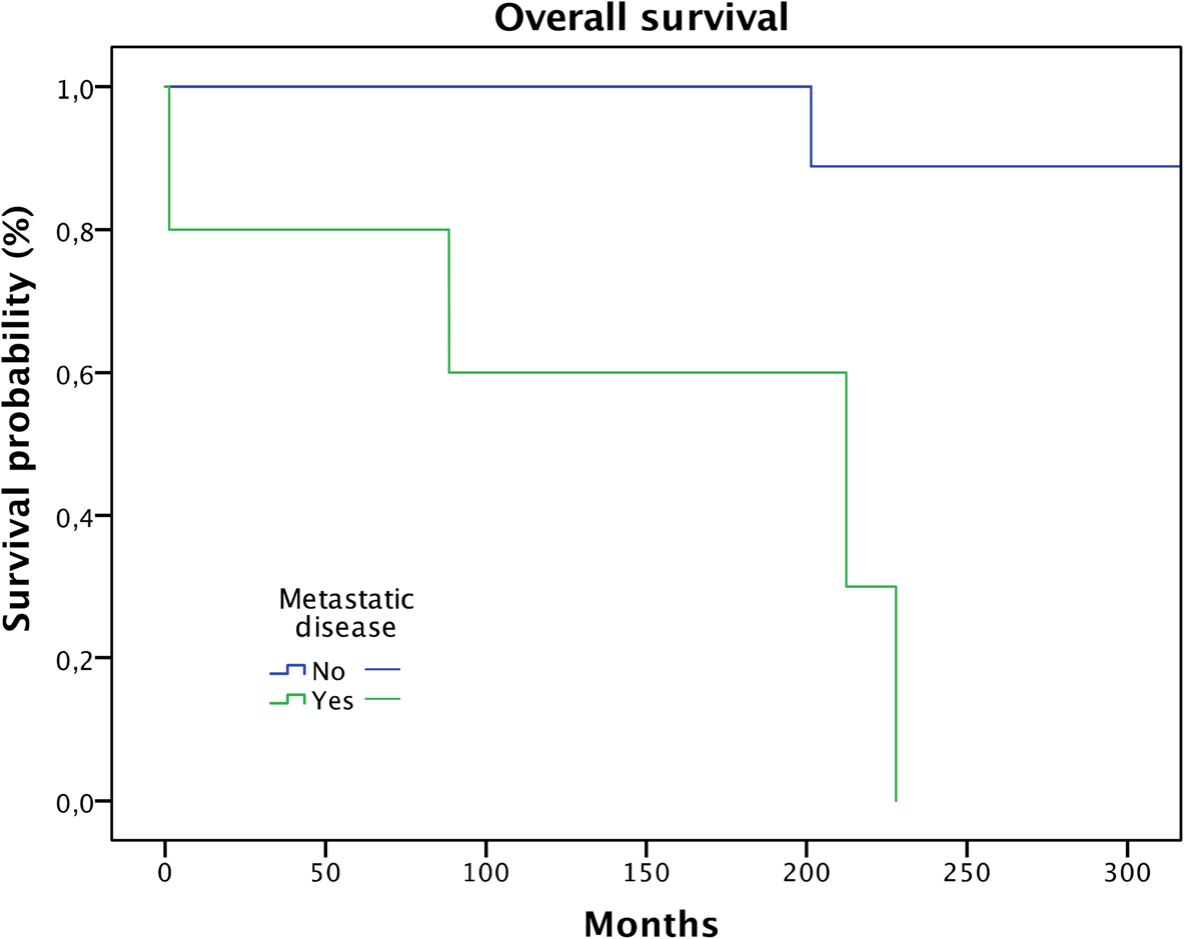
Metastatic disease has a significant negative influence on overall survival (*p* < 0.0001)

Six patients (16%) in our study developed metastases, whereas one patient had an initial spinal metastasis but remained free of disease after resection of that lesion. In five patients (14%), metastatic disease developed during follow-up (four pulmonary, one bone). Mean metastasis-free survival was 86% after 5 years and 75% after 20 years. Four of our patients deceased during the follow-up period (Fig. 3). In the multivariate analysis, there was no correlation between the surgical margins and the location in respect to metastatic disease.

**Fig. 4:**
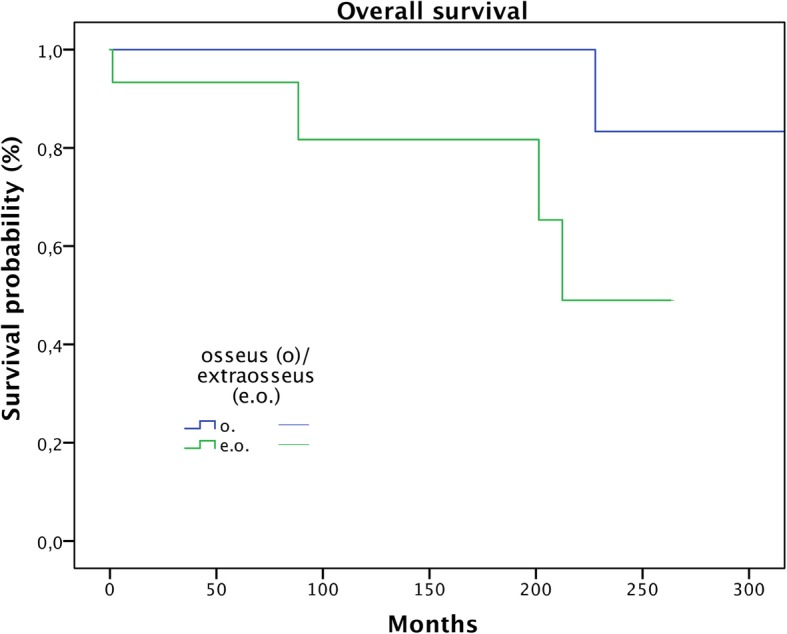
Overall survival of patients in relation to patient age (≤ 50 vs. > 50 years) (n.s.)

**Fig. 5:**
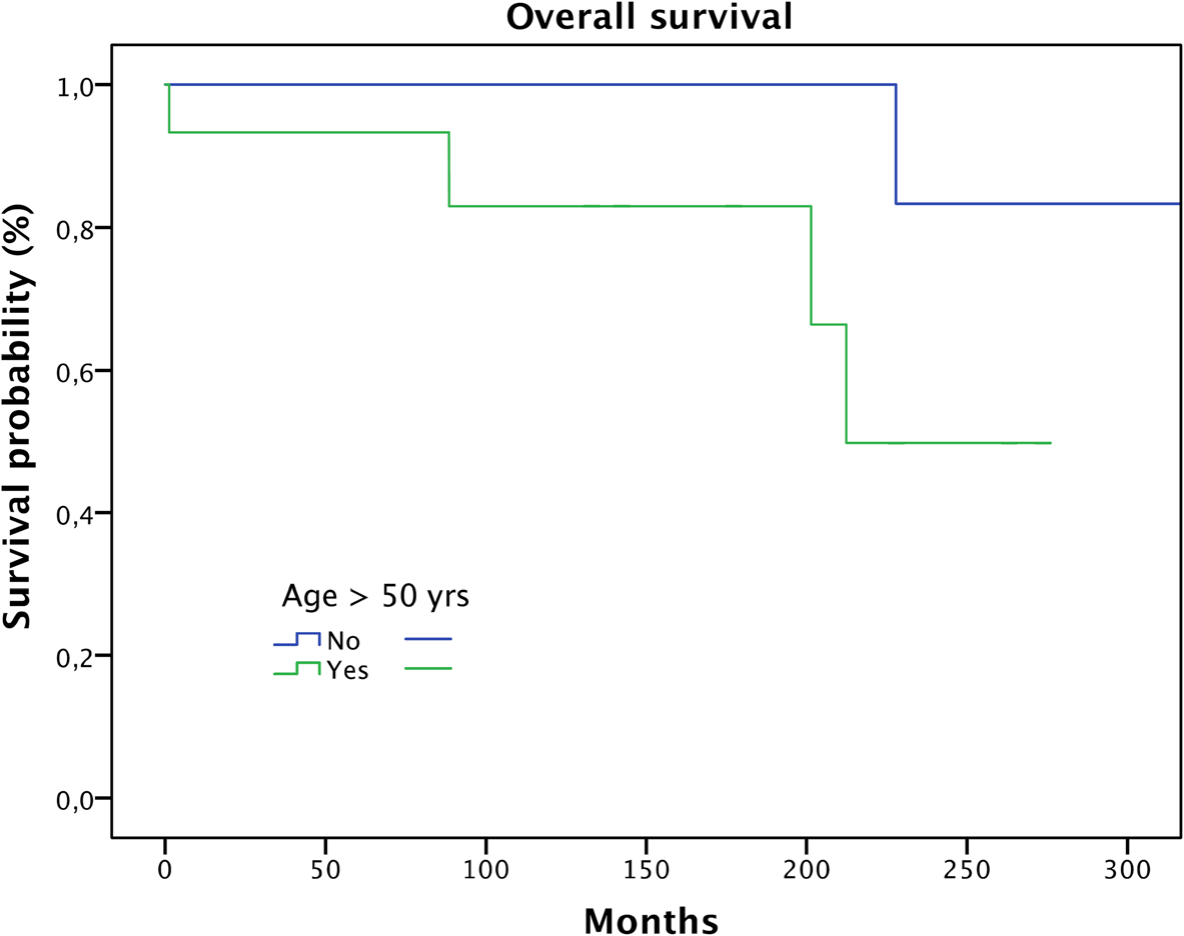
Comparison of overall survival of patients with and without extraosseous tumor involvement (n.s.)

Metastasis proved to be a significant predictive factor for survival (Fig. 3; *p* < 0.0001). Age over 50 showed a trend in respect to worse overall survival, but this failed significance testing (Fig. 4; *p* = 0.078). Only one of the patients (5%) without soft tissue extension died during the follow-up period compared to four patients (27%) with extraosseous infiltration (n.s.) (Fig. 5; *p* = 0.07). Local recurrence and lesion location showed no significance in predicting overall survival.

## Discussion

Grade I chondrosarcoma is generally assumed to be an entity of low malignancy with 5-year survival rates of 90% and more and with little to no metastatic disease [12, 15]. There is, however, no consensus on prognostic factors (i.e., location of the lesion) or the influence of surgical margins (wide resection vs. curettage) and the clinical outcomes in grade I CS.

Although tumor location and patient age have been identified as being strong predictive factors for overall survival in patients with conventional chondrosarcoma in previous publications [16, 17], in our series of patients with low-grade CS, we observed no statistically significant correlation between overall survival and patient age at diagnosis (*p* = 0.078) or tumor location (*p* = 0.238).

A 14% LR rate in our group of patients is similar to previously published numbers of LR in low-grade CS, ranging between 0 and 26% [18–21]. Marginal resection was a significant predictor for LR (*p* = 0.037) in our series. It is known that local recurrence in high-grade chondrosarcoma is associated with poorer outcome, but there is still some debate about whether this is true for low-grade chondrosarcoma as well [22–25]. We were not able to demonstrate a significant correlation between local recurrence and overall survival in this group of patients (*p* = 0.6). Several authors have described a progression of the tumor and the occurrence of distant metastases in association with local recurrence [12, 22, 26, 27]. However, there also are studies that were unable to find such a correlation between LR and MD [23, 24]. In our patients, there was also no correlation between LR and MD with only one patient having both LR and MD. This discrepancy to some of the published literature might be due to the limited mean follow-up of the patients with LR in our group of only 77 months, especially since Schwab et al. described that poor outcomes in patients with LR become significant only beyond 10 years [22].

As mentioned above, there is no consensus on whether or not LR has any significance when it comes to overall survival in low-grade CS. However, many studies have shown that inadequate surgical margins lead to a higher rate of LR, necessitating further surgery with additional risks [10, 23, 24, 27]. Some studies suggest to combine intralesional curettage with adjuvant measures such as the application of poly-methyl-methacrylat (PMMA) or cryosurgery to reduce LR rates [20, 28, 29].

The metastatic potential of low-grade CS is controversially discussed in the literature with rates ranging between 0 and 6% [18, 24, 28, 30]. In our study, one patient already had MD at initial assessment and five patients developed MD during follow-up (16%). A possible reason for this observation could be the longer follow-up of our study with a mean metastasis-free survival of 76 months. Studies which did not describe any MD often report a much shorter follow-up period [31, 32]. However, there are also published studies with a long follow-up and little or no MD as well [9, 20]. This might reflect the difficulty in differentiating between atypical chondrogenic tumors and benign chondromas.

In this study, MD proved to be a significant predictor for a poor outcome (*p* < 0.0001) which is consistent with the findings of other authors. However, we found no significant correlation between the surgical procedure and MD. Interestingly, 2 out of 8 patients with intralesional curettage and 4 out of 16 patients with extraosseous tumor growth developed MD but there was no statistically significant correlation between surgical margins and MD (*p* = 0.45) or extraosseous growth and MD (*p* = 0.21).

Although not statistically significant, the high rate of MD and the higher mortality suggests that extraosseous tumor growth may be a different subtype of CS after all, since it appears to behave differently than regular low-grade CS.

## Conclusion

While the location of the primary tumor is a strong prognostic factor for high-grade CS (i.e., pelvic lesions have a worse prognosis), in this study, there was no significant difference between patients’ outcomes and the tumor location. Marginal resection (R1) was a risk factor for LR, but compared to high-grade CS, there was no significant difference in the overall survival of patients with or without LR. Patients with soft tissue extension of the tumor showed a worse prognosis, but this failed significance testing. MD was more common (16%) than expected and a significant predictor for poor overall survival.

## Data Availability

The datasets used and/or analyzed during the current study are available from the corresponding author on reasonable request.

## Abbreviations

CS: Chondrosarcoma
CT: Computed tomography
G1, G2, G3: Grading according to the French Federation of Cancer Centers’ grading system
LR: Local recurrence
MD: Metastatic disease
MRI: Magnetic resonance imaging
n.s.: Not significant
PMMA: Poly-methyl-methacrylate
R0, R1, R2: Resection margin
